# Multisensory Interactions Influence Neuronal Spike Train Dynamics in the Posterior Parietal Cortex

**DOI:** 10.1371/journal.pone.0166786

**Published:** 2016-12-29

**Authors:** Paul VanGilder, Ying Shi, Gregory Apker, Keith Dyson, Christopher A. Buneo

**Affiliations:** School of Biological and Health Systems Engineering, Arizona State University, Tempe, United States of America; National Eye Centre, UNITED STATES

## Abstract

Although significant progress has been made in understanding multisensory interactions at the behavioral level, their underlying neural mechanisms remain relatively poorly understood in cortical areas, particularly during the control of action. In recent experiments where animals reached to and actively maintained their arm position at multiple spatial locations while receiving either proprioceptive or visual-proprioceptive position feedback, multisensory interactions were shown to be associated with reduced spiking (i.e. subadditivity) as well as reduced intra-trial and across-trial spiking variability in the superior parietal lobule (SPL). To further explore the nature of such interaction-induced changes in spiking variability we quantified the spike train dynamics of 231 of these neurons. Neurons were classified as Poisson, bursty, refractory, or oscillatory (in the 13–30 Hz “beta-band”) based on their spike train power spectra and autocorrelograms. No neurons were classified as Poisson-like in either the proprioceptive or visual-proprioceptive conditions. Instead, oscillatory spiking was most commonly observed with many neurons exhibiting these oscillations under only one set of feedback conditions. The results suggest that the SPL may belong to a putative beta-synchronized network for arm position maintenance and that position estimation may be subserved by different subsets of neurons within this network depending on available sensory information. In addition, the nature of the observed spiking variability suggests that models of multisensory interactions in the SPL should account for both Poisson-like and non-Poisson variability.

## Introduction

Multisensory interactions are critical to both perceptual and motor function. Under certain spatial and temporal constraints, such interactions can result in the fusion of sensory information from different streams, a phenomenon known as multisensory (or multimodal) integration. Such integration is thought to be necessary in part because sensory information is inherently noisy, which can lead to uncertainty in estimating the state of the environment and our own bodies, including the positions and velocities of our limbs. Computational and behavioral studies have demonstrated that combining information from different senses through integration can improve such state estimates [1]. Moreover, several studies have shown that sensory signals are combined in a Bayes-optimal (or nearly optimal) manner, i.e. sensory inputs are weighted according to their relative reliabilities and combined with prior information to maximize the precision of state estimates [2–4].

Despite significant progress in understanding multisensory interactions at the behavioral level, their underlying neural mechanisms remain relatively poorly understood. Until fairly recently, most of what is known comes from studies of a subcortical structure, the superior colliculus (SC), during the interaction of visual, auditory and/or somatosensory inputs. Seminal work by Stein and colleagues established several empirically-derived principles based on studies of the SC, including those of spatial and temporal congruency and the principle of inverse effectiveness [5]. The spatial and temporal congruency principles state that the responses of multisensory neurons will be enhanced in the presence of multiple stimuli provided these stimuli occur close together in space and time, otherwise responses will be suppressed. The principle of inverse effectiveness summarizes the observation that effects at the single cell level are proportionately stronger to the combined presentation of weak stimuli than to the combined presentation of strong stimuli. The combined response to weak stimuli can in some cases be superadditive, i.e. greater than the sum of the responses to individual stimuli, while responses to strong stimuli can actually be less than the sum of the responses to each individual stimulus (i.e. subadditive). Recently, a ubiquitous network-level phenomenon known as divisive normalization has been shown to account for these empirically-derived principles as well as others [6].

As in the SC, multisensory interactions in the cortex are associated with both enhancement and suppression of spiking activity [7–12]. However, the relation between such single-cell level phenomena, larger scale network-level operations and principles of optimal integration derived from theoretical and behavioral studies have only recently been addressed. Ma and colleagues showed that if neural variability in the cortex is assumed to be Poisson-like, Bayes-optimal multisensory integration can conceivably be implemented via a simple linear summation of population responses [13]. This study and others also predict that cortical areas should exhibit largely additive or subadditive responses to multisensory inputs [13, 14]. Interestingly, studies designed to test for optimal integration of visual-vestibular inputs in the dorsal medial superior temporal area showed a predominance of subadditivity [15]. However, although neural variability appears Poisson-like in some parts of the cortex, particularly those situated relatively early in the visual processing stream [16], other areas have been shown to demonstrate more complex patterns of spiking variability under certain task conditions [17–19]. For example, a previous study of the posterior parietal cortex (PPC) found evidence for strong, spatially-tuned, oscillatory spiking during the memory period of an instructed delay reaching task, with spectral power peaking at approximately 25 Hz [18]. Although prevailing computational models of multisensory integration may be insensitive to minor violations of spike-train irregularity, it is not entirely clear how well these models can account for integration in areas and/or tasks where spike trains are highly regular or even oscillatory, such as those described above. As a result, the extent to which neural spike trains are Poisson-like in multisensory areas, particularly during motor tasks, remain an important open question.

The importance of variability in spike timing for multisensory integration has also recently been highlighted by experimentalists, leading to the suggestion that some aspects of integration might even be expressed as a temporal code, e.g. as changes in spike train dynamics and/or oscillatory activity within or across functionally-related neural ensembles [20–22]. For example, multisensory interactions have been associated with altered spike timing in the vestibulocerebellum of the mouse [23] and in the dorsal cochlea nucleus of the guinea pig [24]. In non-human primates, multisensory interactions result in reduced spiking variability [8, 25] and are associated with changes in the phase of ongoing oscillations within a given area [26], as well as changes in inter-areal LFP and spike-field coherence in the gamma band [20, 27]. Regarding the possible role of temporal coding in multisensory state estimation, work in non-human primates suggests that synchronized LFP beta band oscillations serve to bind multiple cortical areas into a large-scale network subserving the maintenance of arm position [28]. Although the roles of different sensory signals were not addressed by Brovelli and colleagues, fMRI work in humans suggests that the different nodes of this putative network could contribute differentially to the maintenance of arm position depending on available sensory information [29].

We recently characterized multisensory interactions in a population of posterior parietal neurons whose activity was recorded during a reach and hold task performed with unimodal (proprioceptive) or bimodal (visual-proprioceptive) sensory feedback [8]. We found that firing rates were largely suppressed under bimodal conditions, consistent with a subadditive interaction of visual and proprioceptive inputs. Average arm endpoint positions and variability in endpoint positions did not differ between unimodal and bimodal conditions however, suggesting that interactions at the single cell level were associated with differences in perceptual and/or motor related variables which were not monitored in these experiments, such as arm configuration [30, 31]. In this study we also found that both across trial and intra-trial variability in spike timing was reduced under bimodal conditions. Given that reduced intra-trial variability can result from the induction or enhancement of oscillatory spiking [17] and that oscillations have been proposed to play an important role in multisensory interactions in the cortex [22], we hypothesized that this population of cortical neurons would show evidence of induced or enhanced oscillatory activity under bimodal conditions. This hypothesis was examined by comparing the power spectra and autocorrelograms of the recorded spike trains between the unimodal and bimodal conditions. Preliminary results of this work have previously been reported in abstract form [32].

## Materials and Methods

### Experimental Subjects and Paradigm

Two rhesus monkeys (Macaca mulatta) were subjects in this study. All animal welfare and experimental procedures were conducted according to the U.S. Public Health Service Policy on Humane Care and Use of Laboratory Animals (Public Law 99–158) and the Guide for the Care and Use of Laboratory Animals (National Academy Press, 1996) and were approved by the Arizona State University Institutional Animal Care and Use Committee. Great care was taken to minimize any pain or discomfort during any medical procedures, using proper anesthesia and analgesia under veterinary care when necessary. All housing, feeding, and environmental social enrichment conformed to institutional standards which are AAALAC International accredited.

**Fig 1:**
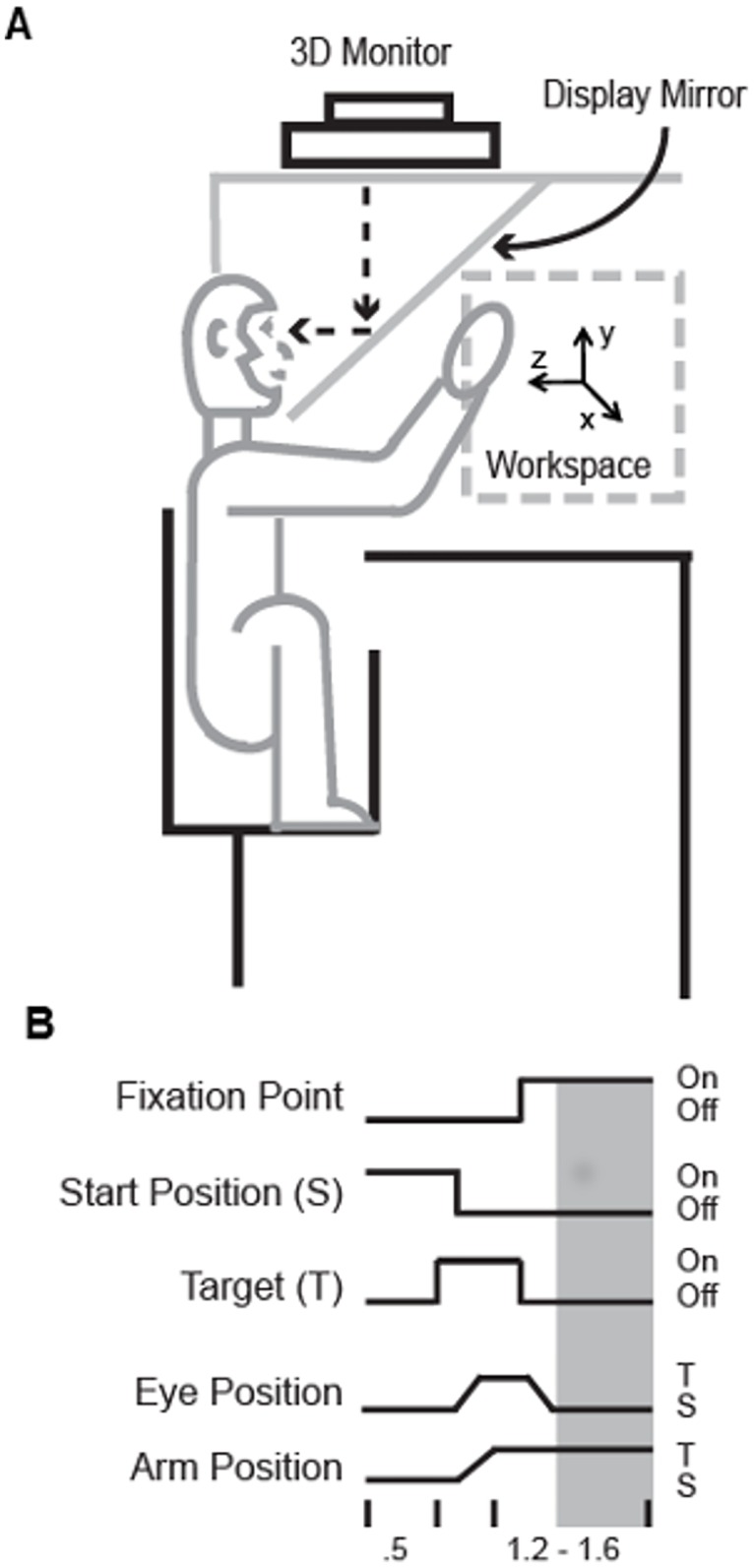
Experimental apparatus and paradigm (Shi et al. 2013). A. Schematic of virtual reality setup. B. Sequence of events on a single trial. Grey rectangle indicates the 800 ms analysis epoch that was the focus of this study. During this time period, animals were required to fixate at the center of the display (S) while maintaining their arm position at the peripherally located targets (T).

The experimental apparatus and paradigm have been described in detail elsewhere [8]. Briefly, two Rhesus monkeys (X and B) were trained to reach to targets in a semi-immersive 3D virtual reality environment and to maintain their arm position at these targets either with or without vision of the endpoint of the arm, which was provided as a virtual sphere (‘arm cursor’). The virtual environment was displayed on a 3D monitor (Dimension Technologies Inc.) and was projected onto a mirror embedded in a metal shield that blocked the real arm from view (Fig 1). Arm position was tracked using an active LED based system (Phoenix Technologies Inc.) and eye position was monitored using a remote optical tracking system (Applied Science Laboratories Inc.).

At the beginning of each trial, an animal first acquired a spherical starting position that was presented at the center of the vertically oriented workspace. After maintaining this position for 500 ms, a spherical green target was presented at one of eight locations arranged in a square (monkey B) or rectangle (monkey X) and up to 6.4–7.1 cm from the center of the workspace. Onset of the target instructed the animals where to reach and also served as the ‘go’ signal. Once the target was acquired it was extinguished after 300–400 msec and a yellow sphere was presented at the center of the workspace, cueing the fixation position. Once the fixation position was acquired a holding period commenced during which time animals continued to fixate the center of the display while maintaining their arm position at the target for 800–1200 msec. During the holding period, animals viewed their arm on half of the trials (bimodal condition) while on the remaining trials vision was prevented by blanking the arm cursor (unimodal condition). In summary, on a given trial animals executed a center-out, reaction-time reach and saccade to one of eight peripheral targets, made a saccade back to a central fixation point, then maintained their arm position at the target for a variable period in the presence of proprioceptive feedback or both visual and proprioceptive feedback. Five (5) trials were performed for each target in each sensory condition, which were pseudorandomly selected.

### Neurophysiological Procedures and Analysis

All experimental procedures were conducted according to the "Principles of laboratory animal care" (NIH publication no. 86–23, revised 1985) and were approved by the Arizona State University Institutional Animal Care and Use Committee. Extracellular recordings (N = 343; 219 from X and 124 from B) were made using varnish-coated tungsten microelectrodes in the superficial cortex of the SPL (area 5), as judged by recording depth and similarity to previous recordings made in this area [18, 33]. Spikes were isolated from the amplified and filtered (600–6000 Hz) signal via a time-amplitude window discriminator (Plexon Inc.). Spike times were sampled at 2.5 kHz.

### Analysis of Neurophysiological Data

Data analyses focused on an 800 ms long section of the holding period beginning 400 ms after target acquisition. Firing rates during this period were compared statistically between conditions using the Mann-Whitney U test using a significance level of α = 0.05. Peristimulus time histograms (PSTHs) of the trial averaged rate were constructed using data from each neuron’s preferred and non-preferred locations during the holding period (based on mean firing rates) and were smoothed with a Gaussian kernel (σ = 50 ms).

We previously quantified the effects of multisensory integration on spiking variability using the Fano factor and coefficient of variation of the interspike intervals [8, 34]. Here, we used the power spectra and autocorrelograms of the recorded spike trains to provide further insights. Power spectra were computed from the spike times on each trial using multitaper spectral methods ([35, 36]; http://chronux.org/). Spectra were computed with a 6.25Hz resolution and nine Slepian data tapers. Single trial spectra were normalized by the mean firing rate during the 800ms window before averaging across trials and cells. The variance of the normalized single cell and population spectra were estimated using a Jackknife procedure that involved leaving out a data taper and creating a distribution of spectral estimates from which the variance was determined. We also computed the variance distribution of individual cells using the autocorrelation function, the time domain counterpart to the spectrum, to look for regularity (oscillations) in the spike trains. Autocorrelograms were computed for each trial by subtracting the shuffle predictor (i.e. the average cross-correlogram of the spike trains from one cell during different trials) from a histogram of the intervals between spikes. The resulting autocorrelogram for all five trials was then normalized by the standard deviation (SD) of the shuffle predictor at each time lag [19, 37] putting them in units of shuffle predictor SDs.

Power spectra and autocorrelograms were used to classify the neural spike trains recorded during the 800 ms holding period using previously published criteria [19, 38]. To classify the spectra from individual cells, we first defined an index A as the average height of the central autocorrelogram bins corresponding to time lags < 5 ms [19]. Spike trains were classified as “bursty” if they exhibited a peak in their power spectrum that exceeded the expected Poisson spectrum by 1 SD in the 5–60 Hz frequency band and had a value of *A* >1 (1 SD higher than the shuffle predictor). We classified spike trains as “refractory” if they exhibited a spectral trough that deviated from the expected Poisson power by 1 SD and had a value of *A* < -1. Spike trains were classified as “Poisson” if their power spectrum showed no deviations from the expected Poisson power and -1 < A < 1. Spike trains were classified as “oscillatory” if their power spectra exceeded the expected Poisson power by 1 SD in the 13-30Hz range and exhibited a value of *A* <1. This sorting paradigm, which is based on previous work [19, 38], utilizes complementary but mathematically equivalent time and frequency domain functions to examine temporal structure in spike trains. Time domain correlation functions are known to suffer from estimation bias and variance, and are particularly susceptible to violations of the non-stationarity assumption [39]. Frequency domain analyses, particularly multitaper spectral methods, greatly reduces the problems associated with estimation bias and variance, and is able to detect subtle changes in temporal structure better than their time-domain counterpart [36]. Owing to these advantages and the ability to construct accurate spectral confidence intervals to assess significant structural features, we also sorted the cells using only the spectral criteria.

Regarding the oscillatory classification, we chose to focus on the 13–30 Hz range for two reasons. First, previous work from the PPC showed strong oscillations in this range during performance of various tasks [18, 19, 40]. In addition, the 13–30 Hz band corresponds to the ‘beta’ band of EEG, LFP, and MEG signals, which has been implicated in the maintenance of sensorimotor state [41], including limb position [28], one of the independent variables in the current study. Although the term “beta oscillations” is typically used to refer to oscillations observed in EEG and other continuous time signals, for convenience we will use this term to refer to rhythmic spiking in the same frequency band. Oscillatory population spectra were computed for both for the preferred and non-preferred locations in each condition. Power in the beta-band was compared between conditions using the Mann-Whitney U test and a significance level of α = 0.05.

## Results

**Fig 2:**
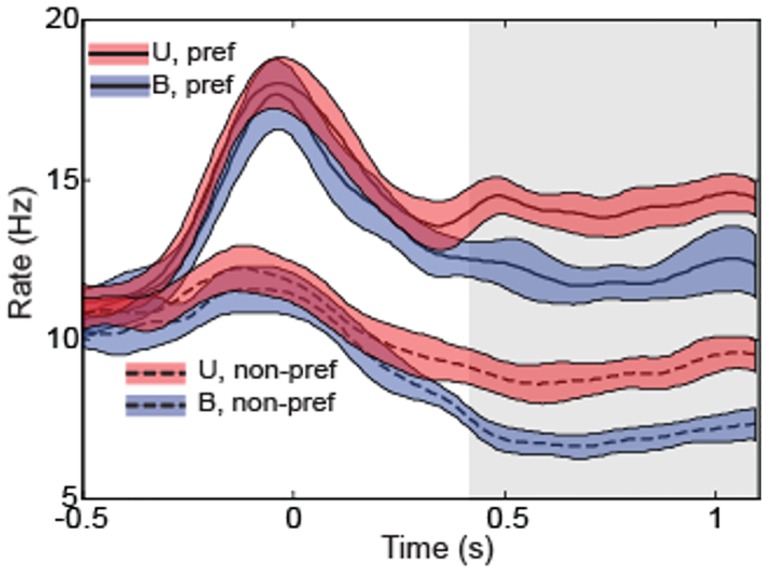
Population PSTHs for all cells (N = 343) in the unimodal (U) and bimodal (B) conditions. Data from the preferred and non-preferred locations are shown aligned to target acquire (Time = 0). Grey rectangle indicates the 800 ms analysis epoch that was the focus of this study. During this time period, animals were required to fixate at the center of a visual display while maintaining their arm position at peripherally located targets.

As described previously, firing rates during the hold period of the bimodal condition were generally suppressed relative to corresponding rates in the unimodal condition. Fig 2 shows PSTHs for both conditions for the population (N = 343), aligned to reach target acquisition (t = 0). Rates were generally indistinguishable between conditions both before and during the movement (i.e. from -0.5s – 0 s), though in both conditions static positional discharge and movement related modulation of activity was clearly evident. During the hold period (0.4s -1.2s), rates were largely stationary but deviated in magnitude between conditions, with activity under bimodal conditions being significantly less than activity under unimodal conditions (p<0.05). Note that the hold period is the only part of the task where the limb feedback conditions differed, i.e. vision of the limb was provided prior to and slightly after arrival at the target and was removed at the time of the saccade to the central fixation position. Given that previous analyses established that neither the mean fixation positions nor the variances of the spatial components of eye position differed between conditions [8], the deviation in firing rates during the hold period can be attributed largely to differences in visual feedback regarding the position of the limb.

**Fig 3:**
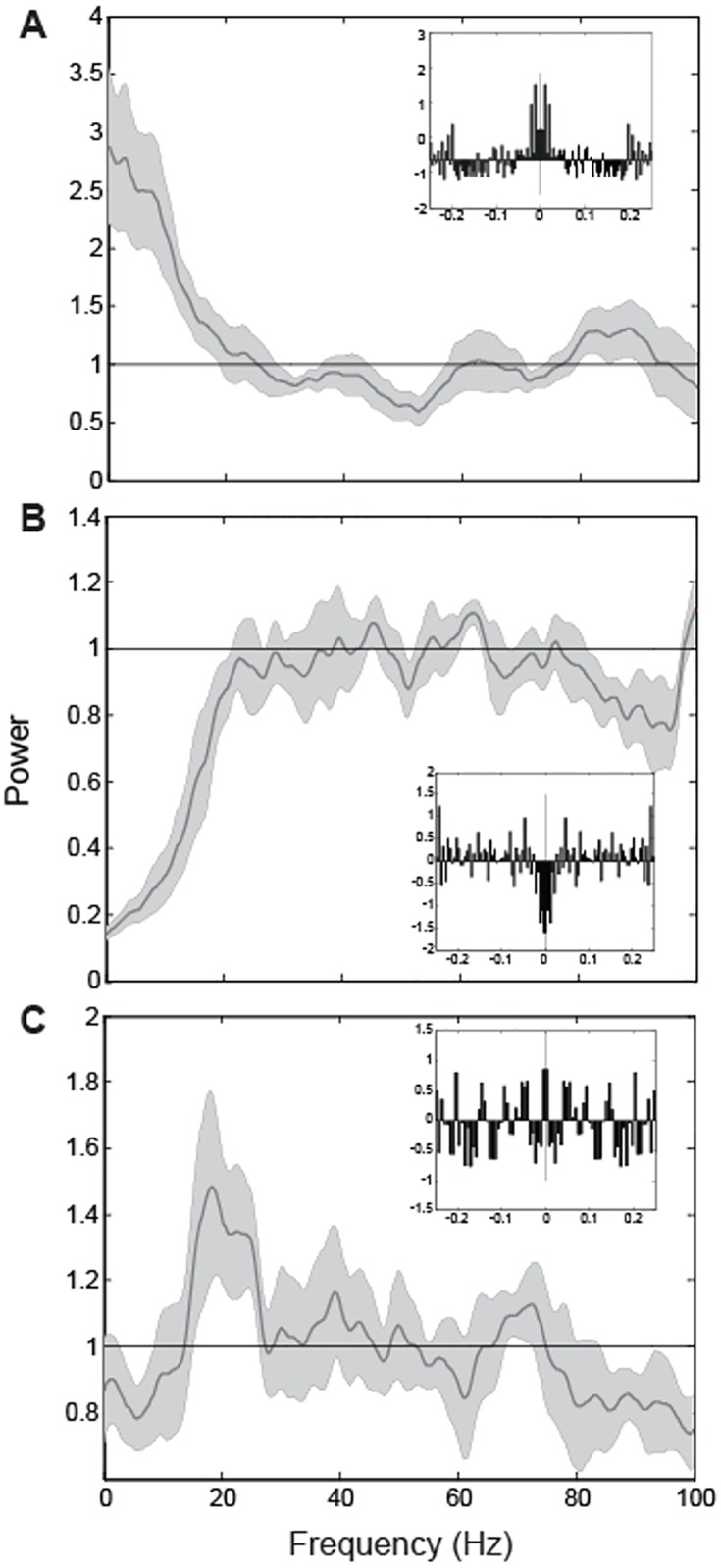
Rate-normalized spike spectra and autocorrelograms (insets) for three example neurons. Data for the preferred location during the holding period of the unimodal condition are shown. Bursty (A), Refractory (B) and Oscillatory (C) neurons are shown. For the autocorrelograms, histogram bars are expressed in units of the shuffle predictor (see Methods for details). Horizontal lines at 1 indicate the power spectrum expected of a Poisson spike train.

Approximately 67% of the neurons (231/343) had a sufficient number of spikes during the holding period in both conditions to compute a spectrum. Fig 3 shows the power spectra and autocorrelograms (insets) for example neurons classified as bursty (A), refractory (B) and oscillatory (C). The spectrum for the bursty neuron shows the enhanced low frequency power (relative to that expected of Poisson spiking) that was representative of this spike train classification as well as an autocorrelogram with the expected peak around zero lag [19]. In contrast, the spectrum for the refractory neuron demonstrates suppressed power at low frequencies (<20 Hz) and an autocorrelogram with a broad central trough. As discussed by Bair et al (1994) and others, spectral suppression is consistent with a relative refractory period, i.e. a decreased likelihood of additional spiking shortly after the occurrence of a spike. The oscillatory neuron (C) also demonstrates a low frequency spectral suppression consistent with a refractory period in addition to broad-band enhancement of spectral power from ~15–30 Hz that peaks at approximately 20 Hz. The autocorrelogram for this cell also shows evidence of temporal structure, with several peaks at relatively equally-spaced time lags on either side of zero lag [19].

The distributions of spike train classifications were highly non-uniform in both the unimodal and bimodal conditions. Table 1 shows the cell classifications for both conditions. In the unimodal condition, few cells were classified as bursty (~4%; 9/231) but refractory spike trains were slightly more common (~17%; 39/231). Oscillatory spike trains were far more common however, with ~38% of the cells (88/231) in the unimodal condition being classified as oscillatory in the 13–30 Hz (beta) frequency band. Notably, no neurons were classified as Poisson-like when both the spectrum and autocorrelation criteria were considered. It should be noted however that the power spectra of some cells were often consistent with oscillatory, bursty, or refractory spike trains but failed to meet the corresponding autocorrelation criteria and were therefore classified as ‘other’. In the unimodal condition, cells in the ‘other’ classification constituted approximately 41% (95/231) of the population of neurons for which a spectrum could be computed.

**Table 1:** Cell classifications in the unimodal and bimodal conditions based on the spectrum and autocorrelation criteria.

	bimodal condition					
unimodal	oscillatory	bursty	refractory	Poisson	other	unclassified
oscillatory	47	1	3	0	37	11
bursty	2	5	0	0	2	3
refractory	3	0	32	0	4	0
Poisson	0	0	0	0	0	0
other	44	1	3	0	47	21
unclassified	0	0	0	0	12	65

Gray boxes indicate the neurons that maintained their classifications between conditions.

‘Unclassified’ refers to cells which did not have a sufficient number of spikes to compute a spectrum in either or both conditions, therefore these cells do not factor into the percentages of spike train classifications reported in the text.

In the bimodal condition, the distribution of spike train classifications was similar to that in the unimodal condition. Here again bursty spike trains were relatively few in number (~3%, 7/231), as were refractory ones (~16%, 38/231). Most of the cells classified as refractory in the bimodal condition were also classified as refractory in the unimodal condition (32/38) suggesting that the spike train dynamics of these neurons were invariant to changes in sensory conditions in this task. This was also true of a large number of the neurons that were classified as oscillatory in the bimodal condition (~42%; 96/231); approximately half of these 96 neurons were also identified as oscillatory in the unimodal condition, again suggesting that their spike train dynamics were invariant to the presence/absence of visual information about the position of the arm. Lastly, as in the unimodal condition, no neurons were classified as Poisson-like in the bimodal condition when both their spectra and autocorrelograms were taken into account.

**Fig 4:**
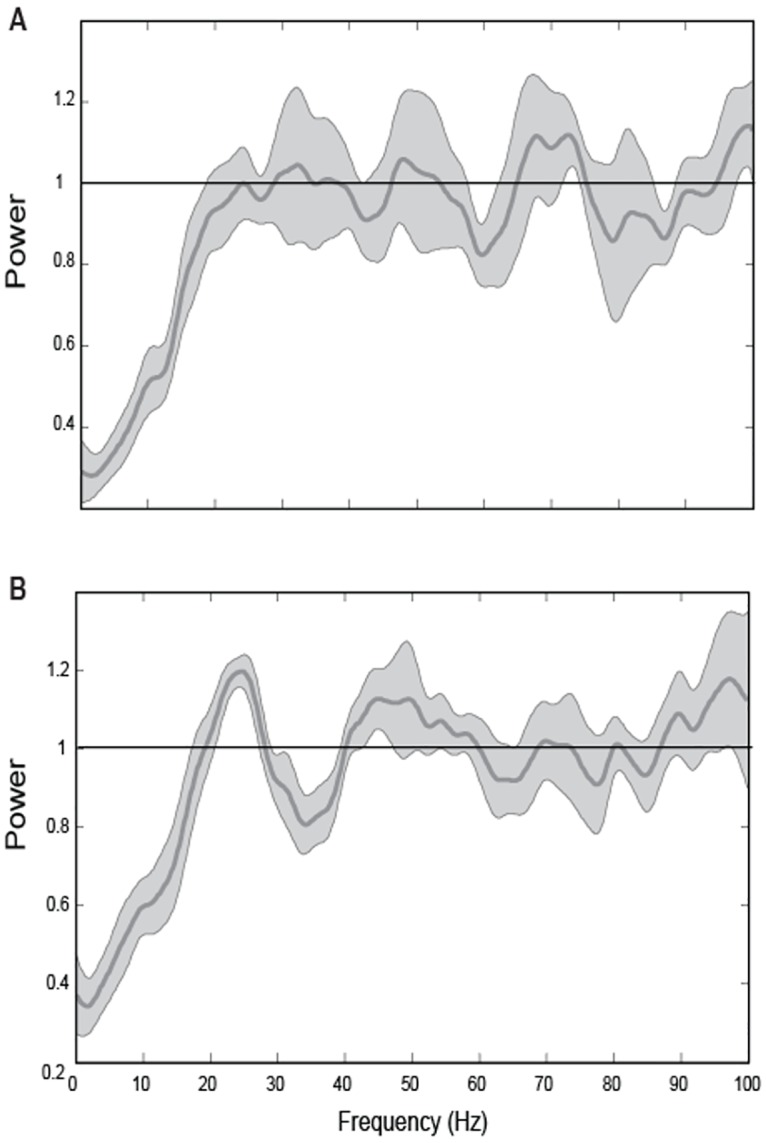
Rate-normalized spike spectra for a single neuron. (A) Bimodal condition and (B) unimodal condition. Horizontal lines at 1 indicate the power spectrum expected of a Poisson spike train.

Although many neurons retained their spike train classifications between conditions, the spike train dynamics of a substantial number of ‘other’ neurons (100/231; 43%) changed. The most commonly observed difference in spike timing was the presence of beta oscillations in one condition but not the other. Fig 4 shows spectra for one of these neurons in both conditions. On trials where both visual and proprioceptive feedback was provided (A), the spectrum exhibited low frequency suppression up to ~ 20 Hz, consistent with refractoriness, but at frequencies greater than 20 Hz the spectrum showed only very small deviations from that expected of a Poisson process. On trials where only proprioceptive feedback was available (B), low frequency suppression up to ~20 Hz could also be observed. However, this was followed by a substantial peak in the spectrum that was centered at approximately 25 Hz, i.e. in the beta band of frequencies. It’s important to reiterate that unimodal and bimodal trials were interleaved in this task. Thus, on a trial by trial basis beta-band oscillations would alternately appear or disappear from the spike trains of this cell and many others, and this was directly related to the nature of the feedback the animal was receiving.

**Fig 5:**
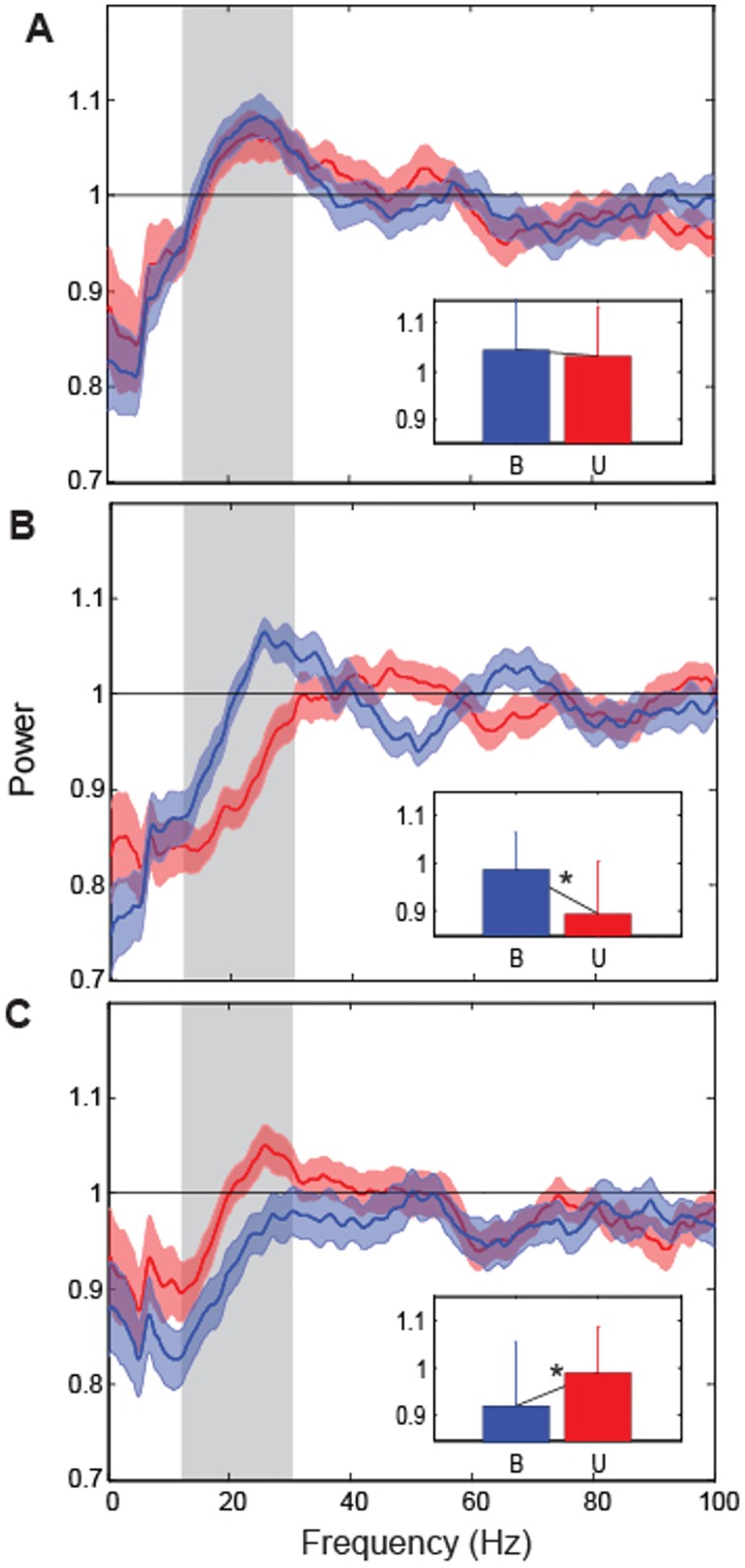
Population spike spectra for oscillatory cells in the unimodal and bimodal conditions. Spectra for the preferred location for neurons classified as oscillatory in both conditions (A; N = 47), oscillatory in the bimodal condition only (B; N = 49) and oscillatory in the unimodal condition only (C; N = 41).

Approximately equal numbers of neurons showed evidence of beta oscillations in the unimodal condition, bimodal condition, or both conditions. Fig 5 shows population spectra for the preferred location for neurons classified as oscillatory in both conditions (A), oscillatory in the bimodal condition only (B) and oscillatory in the unimodal condition only (C). In Fig 5A a broad-band peak centered at ~25 Hz is apparent in the spectra for both conditions. Power in the beta-band (inset) did not differ significantly between conditions for this group of cells (p = 0.56). Thus, the spike train dynamics of these neurons were invariant to changes in the sensory conditions in this task. In contrast, in Fig 5B a similar spectral peak in the beta-band can be observed for the bimodal condition but not the unimodal condition; for this group of neurons power in the beta-band did in fact differ significantly between conditions (p<0.05). Although a smaller peak in the beta-band is apparent for the unimodal condition in Fig 5C, power also differed significantly between conditions for this group of cells (p<0.05). Thus, for the subsets of neurons shown in Fig 5B and 5C, beta-band oscillations were apparent in one set of sensory conditions but not the other.

It should be noted, that when sorting the neurons using only the power spectrum criterion, none were classified as “other,” indicating that the autocorrelogram-based criteria served only to exclude cells from one of the classifications. The most apparent change when sorting cells based solely on their spectra was a much more even distribution of cells. Aside from a single bursty cell in the bimodal condition, all other classifiable cells fell into either the oscillatory or refractory categories and did so in similar proportions (Table 2). In the bimodal condition, 54% of neurons were classified as oscillatory (125/231) and 45% (105/231) were refractory. For the unimodal condition the proportions were nearly equivalent, with 49% (114/231) being oscillatory and 50% (116/231) being refractory. There was an increase in the number of cells that were oscillatory in both conditions (76/231, 33%) relative to those that were oscillatory only in the unimodal (38/231, 16%) or bimodal (49/231, 21%) conditions, but the majority of cells that had been previously classified as “other” were reclassified as refractory in one or both conditions.

**Table 2:** Cell classifications in the unimodal and bimodal conditions, based on the spectrum criteria only.

	bimodal condition					
unimodal	oscillatory	bursty	refractory	Poisson	other	unclassified
oscillatory	76	1	38	0	0	24
bursty	0	0	0	0	0	0
refractory	49	0	67	0	0	11
Poisson	0	0	0	0	0	0
other	0	0	0	0	0	0
unclassified	7	0	5	0	0	65

## Discussion

Although several studies have shown that the interaction of different sensory processing streams alters neuronal firing rates in the primate brain, the relationship between such interactions and action potential timing has remained relatively unexplored. Based on previous work [18, 22, 28, 29] we hypothesized that SPL neurons would show evidence of oscillatory activity in the beta band during the sustained maintenance of arm position, and that these oscillations would be enhanced under bimodal conditions. Given that more regular (e.g. oscillatory) spiking leads to reduced spike timing variability [17], enhanced spiking oscillations under bimodal conditions would also be consistent with our previous findings of reduced intra-trial spiking variability under bimodal conditions [8]. Although oscillatory spiking was commonly observed here, we did not find evidence that these oscillations were consistently enhanced under bimodal conditions. Instead some neurons fired rhythmically in the beta band under only one set of sensory conditions (unimodal or bimodal), while others did so under both conditions. The results suggest that the SPL may belong to a putative beta-synchronized network for arm position maintenance [28] and that position estimation could be subserved by different subsets of neurons within this network depending on available sensory information. In addition, the nature of the observed spiking variability observed in this study suggests that computational models of multisensory interactions in the SPL and elsewhere should account for both Poisson-like and non-Poisson variability.

### Behavior

In the experimental sessions described here, animals performed a reach and hold task while receiving either unimodal (proprioceptive) or bimodal (visual-proprioceptive) sensory feedback [8]. Despite consistent difference in firing rates between the two conditions (described below; see also Fig 2), average arm endpoint positions and variability in endpoint positions did not differ between unimodal and bimodal conditions. We have also previously reported no differences in average eye position or variability in eye position during this task [8]. No difference in arm endpoint position was an expected consequence of our experimental design. In this initial exploration of multisensory interactions we wanted to ensure that any differences in firing rates that were observed did not reflect the fact that animals held their limbs at slightly different positions in the two tasks and were instead due to the different visual conditions. As a result, animals were trained to maintain their arm position within a very tight behavioral window in both tasks. However, this behavioral constraint also resulted in endpoint variability being roughly equivalent between the two conditions. It’s currently unclear how to interpret difference in firing rate during multisensory interactions without a concomitant change in behavior. It should be noted however that other studies exploring the neural correlates of multisensory integration have also not observed a concomitant change in perceptual or action-related variables mainly because such variables were not monitored. For example, work by Graziano and colleagues have previously shown differences in firing rates with and without arm vision in a task where the animal was not required to move or make a perceptual judgement [11]. Since it is impossible to monitor all possible behavioral variables in a single experiment, the most logical explanation for the observation of a neural correlate without a corresponding behavioral correlate is a perceptual or behavioral change that is not observed simply because it is not monitored. Given the known sensitivity of SPL neurons to differences in arm configuration [30, 31], the most likely explanation for our results is that animals used either a slightly different arm configuration while maintaining their position with and without visual feedback, or they used roughly the same configuration but exhibited differing degrees of variability in configuration between conditions. Additional experiments will be necessary to distinguish between these and other potential explanations for the changes in neural activity reported here and elsewhere.

### Effects of Multisensory Interactions on Firing Rates

As described previously by Shi et al (2013), firing rates were suppressed under bimodal conditions, consistent with a subadditive interaction of visual and proprioceptive inputs. Early work by Stein and colleagues in the SC described additive, subadditive and superadditive responses to multisensory interactions depending on factors such as spatial and temporal congruency. More recent experimental work however suggests that superadditive responses are less common than initially believed, being observed generally for weak responses to unimodal stimuli [42, 43]. This finding is more in line with computational modeling studies employing a probabilistic population coding framework which predicts that cortical areas should exhibit largely additive or subadditive responses [13, 14]. Interestingly, recent studies of visual-vestibular interactions under conditions designed to test for optimal integration of these inputs showed a predominance of subadditivity in the dorsal medial superior temporal area [15]. Similarly, studies of other cortical regions employing different experimental paradigms [12, 44–46] have reported substantial degrees of multisensory suppression (and therefore subadditivity). Although the significance of this relatively ubiquitous suppression is still not entirely clear, it is noteworthy that some studies have provided evidence that suppression of spiking and reduced spiking variability resulting from multisensory integration is associated with greater encoded stimulus information and greater population decoding accuracy [8, 25].

### Spike Train Dynamics

A relatively large number of neurons in this study could not be classified as Poisson, bursty, refractory, or oscillatory and were therefore classified as ‘other’. This is due in part to the fact that the employed spike train classification scheme relies on meeting two separate criteria, one based on the power spectrum and the other on the autocorrelation function. Many cells exhibited power spectra that were consistent with bursty, refractory or oscillatory spike trains but did not meet the corresponding autocorrelation criteria or vice-versa. Of the cells with internally consistent spectra and autocorrelations, the vast majority were classified as oscillatory in the beta-band in one or both conditions. This preponderance of oscillatory spiking in the beta-band is consistent with other studies of the PPC using different tasks and behavioral epochs [18, 19]. It should be noted that virtually all of the oscillatory spike trains were accompanied by a spectral trough at low frequencies. Although somewhat nonintuitive, this suppression is consistent with a refractory period, i.e. a decreased probability of spiking shortly after the occurrence of a spike [16]. A substantial number of cells in our population also showed evidence of refractoriness without accompanying oscillations, also consistent with previous investigations [16, 18, 19, 38].

Although some neurons demonstrated power spectra that appeared Poisson-like, none were classified as Poisson when the corresponding autocorrelograms were considered. Although Poisson-like spiking has been reported in several subdivisions of the PPC, the percentage of cells classified as such appears to be task-, epoch-, and area-dependent [17, 19]. The relative dearth of Poisson-like spike trains reported here has important implications for computational models of multisensory integration. That is, recent studies have demonstrated that Bayes-optimal multisensory integration can be implemented as a simple linear summation of cortical activity assuming that neural variability in the cortex is approximately Poisson-like [13]. The present findings however reinforce previous studies that this assumption does not hold for all cortical regions. Although the model of Ma et al. is at least somewhat insensitive to violations of Poisson-like variability, it is not clear how well the model can accommodate neural variability which is highly regular, as observed here. As a result, it may be necessary for future modeling efforts to account for both Poisson-like and non-Poisson neural variability.

### Temporal Coding of Multisensory Information for State Estimation

Although the basic premise of temporal coding, i.e. that information is encoded not only in spike rates but also in the precise timing of action potentials and/or ongoing neuronal (LFP) oscillations, has been postulated to play a role in phenomena such as feature binding, its relevance to multisensory interactions has been addressed only relatively recently [22]. Some of the strongest evidence in support of this idea comes from analyses of neural data obtained from non-human primates engaged in tasks involving somato-auditory and audio-visual interactions. For example, interaction of somatosensory and auditory inputs has been shown to result in changes in the relative phase of ongoing oscillations within primary auditory cortex [26]. Similarly, audio-visual interactions are associated with enhanced LFP and spike-field gamma coherence between auditory cortex and the superior temporal sulcus [20, 27]. Regarding changes in spiking, multisensory interactions have been shown to alter spike timing in the vestibulocerebellum of the mouse [23], in the dorsal cochlea nucleus of the guinea pig [24], and in the cortex of non-human primates engaged in tasks involving audio-visual and visual-proprioceptive interactions [8, 25]. The current findings extend those of Shi et al (2013) by showing that changes in spike timing can manifest differently in different subpopulations within a given cortical area, with potentially important implications for transcortical representations of perceptual and motor-related phenomena.

The present findings are consistent with the idea that the SPL is involved in estimating the state of the arm, as suggested by clinical and functional imaging studies in humans [29, 47–49] as well as neurophysiological studies in monkeys [8, 11, 30, 31, 33, 50, 51]. Previous work in monkeys, has also shown that static maintenance of arm position involves a beta-synchronized cortical network that includes the somatosensory cortex, inferior parietal lobule, and primary motor cortex [28]. The strong beta oscillations observed in the present study suggest that the SPL is also a part of this network and support the idea that beta oscillations serve to functionally link ensembles of neurons in cortical and subcortical regions that are believed to be involved in the maintenance of current motor state [21, 22, 41, 52].

Contrary to our stated hypothesis we did not find evidence that beta oscillations were consistently enhanced under bimodal conditions. Instead roughly equal numbers of neurons fired rhythmically in the beta band under only one set of sensory conditions (unimodal or bimodal) or both conditions and in the latter case, beta band power was the same magnitude in the two conditions. The fact that the beta oscillations of some cells were modulated by the presence/absence of visual input at all suggests that these oscillations are associated not only with sustained muscle activation and somatosensory feedback, as has previously been suggested [53], but also with ongoing visual feedback used for state estimation. However, the lack of a consistent bias in the population raises the question as to the functional significance of these findings. One possible interpretation is that different subsets of neurons exhibiting beta oscillations during proprioceptive, visuo-proprioceptive, and both conditions, reflects the differential recruitment of partially overlapping neural ensembles devoted to these respective modes of sensorimotor control during the maintenance of arm position [54]. The idea that control mechanisms for the arm may differ in the presence and absence of vision is supported by numerous psychophysical studies in humans [55–60]. In further support of this idea, functional imaging studies in humans indicate that the recruited nodes of a cortical network for limb position vary depending on available sensory information. For example, in the absence of vision, interaction of tactile and proprioceptive inputs has been shown to involve the right PPC, whereas in the presence of vision, interaction recruits a network of areas including the parietal and premotor cortices [29]. Validating this hypothesis more directly would require recording from single neurons in multiple nodes of the state estimation network simultaneously and looking for evidence of functional coupling between subsets of neurons only under a prescribed set of sensory conditions. Such manipulations are intended to be the focus of future planned series of studies.
